# Dietary Addition With *Clostridium butyricum* and Xylo-Oligosaccharides Improves Carcass Trait and Meat Quality of Huanjiang Mini-Pigs

**DOI:** 10.3389/fnut.2021.748647

**Published:** 2021-11-05

**Authors:** Qiaoli Cai, Chengjun Hu, Wu Tang, Huijiao Jiang, Meimei Geng, Xingguo Huang, Xiangfeng Kong

**Affiliations:** ^1^Hunan Provincial Key Laboratory of Animal Nutrition Physiology and Metabolism Process, Institute of Subtropical Agriculture, Chinese Academy of Sciences, Changsha, China; ^2^College of Animal Science and Technology, Hunan Co-Innovation Center of Animal Production Safety, Hunan Agricultural University, Changsha, China; ^3^Tropical Crops Genetic Resources Institute, Chinese Academy of Tropical Agricultural Sciences, Haikou, China

**Keywords:** carcass trait, *Clostridium butyricum*, growth performance, Huanjiang mini-pigs, meat quality, xylo-oligosaccharides

## Abstract

This study was conducted to investigate the effects of dietary addition with *Clostridium butyricum* (CB) and xylo-oligosaccharides (XOS) on growth performance, carcass trait, and meat quality of pigs. A total of 128 Huanjiang mini-pigs with an initial body weight of 9.5 ± 0.1 kg were randomly assigned to one of four groups. The pigs in control (Con) group were fed a basal diet and those in the experimental groups were fed the basal diet supplemented with 0.05% CB (CB group), 0.02% XOS (XOS group), or 0.05% CB + 0.02% XOS (CB + XOS group). Eight replicate pens were used per group with four pigs per pen. On days 28, 56, and 84 of the trial, the growth performance, carcass trait, and meat quality were evaluated. The results showed that dietary CB addition decreased (*p* < 0.05) the average daily gain and increased (*p* < 0.05) the ratio of feed intake to body weight gain at day 28 of the trial; CB, XOS, and CB + XOS addition increased (*p* < 0.05) the backfat thickness at day 84 of the trial compared with the Con group. Dietary CB, XOS, and CB + XOS addition increased (*p* < 0.05) the pH_45min_, while decreased (*p* < 0.05) the marbling score at day 28 of the trial compared with the Con group. Dietary CB + XOS addition increased (*p* < 0.05) the contents of Ala, Arg, Asp, Gly, His, Leu, Lys, Met, Phe, Ser, Thr, Tyr, and Val in muscle at day 56 of the trial. At day 84 of the trial, dietary CB addition increased the contents of nonessential amino acid (NEAA), total amino acid (TAA), and monounsaturated fatty acid (MUFA), while decreased (*p* < 0.05) the percentage of C20:1 in muscle compared with the Con group. Collectively, dietary addition with 0.05% CB and 0.02% XOS could not alter the growth performance, but increase carcass trait, meat quality, and muscular nutrient contents in Huanjiang mini-pigs.

## Introduction

The pork is one of the most important animal-derived foods for human consumption. However, less skeletal muscle yield is one of the major problems in swine production (1). Consumers increasingly focus on the relationship between nutrition and health and show a preference for high-quality meat products (2). Consequently, improving the meat yield and quality has naturally become a hot issue in animal nutrition research. Meat quality can be improved by nutrition strategy (3). Therefore, developing a novel feed strategy is an urgent necessary for swine production.

Previous study showed that there is a potential link between the gut microbes and body fat deposition in animals (4). For instance, microbiota transplants from obese mice induced obesity in germ-free mice (5). In addition, intestinal microbes present important impacts on the physiological activities and health of the host (6). Therefore, modulation of gut microflora might be a feasible mean to improve meat quality. Recently, *Clostridium butyricum* (CB) and xylo-oligosaccharides (XOS) have attracted growing interest. Dietary addition with these additives could provide nutrients for probiotic *Bifidobacteria* and *Lactobacilli* and preferentially stimulate the growth or activity of indigenous bacteria in the gut, thus maintaining intestinal microflora balance and promoting animal growth (7, 8). In addition, dietary CB could produce short-chain fatty acids (SCFAs) (9) and XOS could be utilized by the bacteria to produce SCFAs (10). The SCFAs play a vital role in maintaining gut integrity and regulating glucose homeostasis, lipid metabolism, appetite, and immune function (11). Therefore, the CB and XO as effective feed additives have been widely used in swine production. However, the interactive effects of these additives on the growth performance, carcass trait, and meat quality of pigs are still unclear.

Huanjiang mini-pig is a local miniature pig breed and is well known for its high-quality meat. The slaughter weight required by the different consumption methods is different. For example, roast pork generally selects 7.5–10 kg of mini-pigs, while processing bacon requires a larger body weight of mini-pigs. However, this breed is mainly fed with corn, wheat bran, rice bran, and low-quality green roughage (12), which are unfavorable to the growth potential and meat quality of mini-pigs. Therefore, the development of effective feed additives is of great significance for improving their meat quality. We hypothesized that dietary addition with both CB and XOS may synergistically improve the growth performance and meat quality of pigs. This study was conducted to test this hypothesis by determining growth performance, carcass trait, and meat quality of Huanjiang mini-pigs with different body weights (ages) after dietary CB and XOS addition and provided a novel nutrition intervention strategy for meat quality improvement.

## Materials and Methods

### Animals, Diets, and Treatments

A total of 128 Huanjiang mini-pigs with an initial body weight (BW) of 9.5 ± 0.05 kg were randomly assigned into one of four groups. Each group consisted of eight replicate pens with four pigs per pen (male: female = 1:1). The pigs in Con group were fed a basal diet and those in the experimental groups were fed the basal diet supplemented with 0.05% CB (CB group), 0.02% XOS (XOS group), or 0.05% CB + 0.02% XOS (CB + XOS group). A corn and soybean meal-based diet refers to the recommendations of the Chinese National Feeding Standard for Swine (2004) and premix refers to the National Research Council (NRC) recommended requirements (2012) for growing-finishing pigs (Table 1). The CB was provided by the Shandong Baolai-Leelai Bio-Industrial Corporation, Ltd., Taiwan, China and contains ≥1 × 10^9^ colony-forming unit (CFU) of viable CB per gram; the XOS was provided by the Shandong Longlive Bio-Technology Corporation, Ltd., Dezhou, China and the content of main components (including xylose, xylotriose, and xylotetraose) is ≥35%. The doses of CB and XOS in the diets were 0.05 and 0.02%, respectively.

**Table 1:** Compositions and nutrient levels of basal diets (air-dry basis) (%).

**Ingredients**	**Prophase nursery diet**	**Anaphase nursery diet**
Corn	55.00	58.00
Soybean meal	22.00	17.65
Wheat bran	10.63	12.05
Rice bran	8.37	8.30
Premix^a^	4.00	4.00
Total	100.00	100.00
**Nutrient levels** ^**b**^		
Digestible energy (MJ/kg)	13.48	13.38
Crude protein	16.13	14.70
Crude fiber	14.67	14.96
Calcium	0.70	0.69
Phosphorus	0.38	0.38
Lysine	0.88	0.78
Methionine + Cysteine	0.50	0.47
Threonine	0.51	0.46

a *Premix provided the following for per kilogram of the diet: Vitamin A 4,000 IU, Vitamin D3 1,200 IU, Vitamin E 40 IU, Vitamin K3 0.8 mg, Vitamin B1 1.6 mg, Vitamin B2 1.6 mg, Vitamin B6 1.2 mg, Vitamin B12 16 μg, biotin 0.08 mg, pantothenic acid 6 mg, niacin 14 mg, choline chloride 300 mg, Cu 127 mg, Fe 171.6 mg, Zn 116 mg, Mn 43 mg, K 0.34 mg, I 0.26 mg, Co 0.14 mg, and Se 0.16 mg*.

b *Nutrient levels were calculated values*.

After 3-day adaptation period, the pigs were housed in pens and had free access to their diets and water. The feeding trial lasted for 84 days and the animals were fed a prophase nursery diet (from 1 to 56 days) or an anaphase nursery diet (from 57 to 84 days).

### Growth Performance Determination

The BW of pigs was weighed at the beginning and the termination of the trial for calculating average daily gain (ADG). Feed intake was weighed daily to calculate the average daily feed intake (ADFI). The feed efficiency was determined by using the F:G ratio based on the feed intake and BW.

### Sample Collection

On days 28, 56, and 84 of the feeding trials, pigs (eight pigs per treatment with one pig per pen) with average BW were selected for sample collection. The diet was removed 12 h before slaughtering. All the animals were sacrificed under the commercial conditions by using electrical stunning (120 V, 200 Hz) and exsanguination. After the head, feet, tail, and internal organs were removed, the carcass was weighed and recorded to calculate the carcass yield (carcass weight/preslaughter weight × 100%) and then cut into two parts longitudinally. After slaughtering, fresh *longissimus dorsi* (LD) muscle between the sixth and seventh ribs on the right side of carcass was immediately collected for meat color, pH value, and marbling score determination, and then stored at −20°C for analysis of chemical composition.

### Carcass Trait Measurement

The backfat thickness, skin thickness, and loin eye area between the sixth and seventh ribs on the left side carcass were measured by using a Vernier caliper. The left side of carcass was weighed and then dissected into skin, skeletal muscle, fat, and bone. Carcass composition was calculated by dividing the tissue weight by the carcass weight (13).

### Meat Quality Assessment

Meat quality was estimated by using meat color, pH value, and marbling score. The objective color of fresh meat was evaluated by using the Konica Minolta Chroma Meter (CR410, Konica Minolta Sensing Incorporation, Tokyo, Japan) with a 50-mm aperture, Φ50 mm measurement area, and D65 light source. The a^*^ (redness), b^*^ (yellowness), and L^*^ (lightness) values were recorded, respectively. The pH_45min_ postmortem was determined by using a handheld pH meter (Russell CD700, Russell pH Ltd., Fife, UK) by inserting the electrode into the core of the muscle parallel to muscle fibers (14).

### Chemical Analysis of Skeletal Muscle

After excess fat and fascia were removed, muscle samples were cut into thin slices, placed in a tray, dried by using a vacuum freeze dryer (50ND, Scientz, Ningbo, China), and then ground into powder by using an electric shredder. The crude protein (CP) and intramuscular fat (IMF) of the muscle samples were determined by using Kjeldahl and Soxhlet extractions with petroleum ether, respectively, according to the Association of Official Analytical Chemists methods. The hydrolyzed amino acid (AA) contents in muscle samples were determined as described previously (15) by using an AA analyzer (L8900, Hitachi, Tokyo, Japan). The fatty acid (FA) composition of LD muscle was determined according to the previously described method (16) by using a gas chromatographer (7890A, Agilent Technologies, California, USA) equipped with a flame ionization detector and a CP-Sil 88 fused silica open tube capillary column (100 × 0.25 nm; Varian Chrompack, USA). The individual FA was quantified according to the peak area and expressed as a percentage of total FA.

### Statistical Analysis

Data were statistically analyzed by the one-way ANOVA by using the General Linear Model (GLM) procedure of the Statistical Analysis System (SAS) 9.2 software (Institute Incorporation, Cary, North Carolina, USA). When a significant interaction effect was found, the Duncan's multiple comparison test was performed. Replicate was considered as the experimental unit. Data were expressed as treatment means with their pooled SEM and the differences between means were considered as statistically significant at *p* < 0.05.

## Results

### Growth Performance

As shown in Table 2, no differences (*p* > 0.05) were observed in the final BW and ADFI among the four treatments. Compared with the Con group, dietary CB addition decreased the ADG and increased the F:G ratio at day 28 of the trial (*p* < 0.05). In addition, the F:G ratio in the CB + XOS group was lower (*p* < 0.05) during days 1–84 of the trial compared with the CB group.

**Table 2:** Effects of dietary addition with *Clostridium butyricum* (CB) and xylo-oligosaccharides (XOS) on growth performance of Huanjiang mini-pigs.

**Items**	**Con group**	**CB group**	**XOS group**	**CB + XOS group**	**SEM**	***p* value**
Initial BW, kg	9.49	9.49	9.49	9.49	0.045	0.84
Finial BW, kg	36.91	36.39	37.40	37.23	0.500	0.76
**Days 1–28**						
ADFI, kg/d	0.52	0.51	0.52	0.50	0.06	0.39
ADG, kg/d	0.19^a^	0.16^b^	0.20^a^	0.19^a^	0.05	<0.01
F/G ratio, kg/kg	2.65^b^	3.16^a^	2.52^b^	2.73^b^	0.19	<0.01
**Days 29–56**						
ADFI, kg/d	1.20	1.20	1.21	1.18	0.08	0.60
ADG, kg/d	0.38	0.43	0.39	0.38	0.08	0.23
F/G ratio, kg/kg	2.86	2.88	3.01	3.12	0.21	0.45
**Days 57–84**						
ADFI, kg/d	1.57	1.57	1.58	1.53	0.10	0.64
ADG, kg/d	0.39	0.34	0.38	0.39	0.08	0.19
F/G ratio, kg/kg	4.04	4.68	4.31	4.11	0.25	0.08
**Days 1–84**						
ADFI, kg/d	1.10	1.09	1.10	1.07	0.07	0.39
ADG, kg/d	0.32	0.31	0.33	0.32	0.05	0.52
F/G ratio, kg/kg	3.43^ab^	3.57^a^	3.28^b^	3.32^b^	0.15	0.02

*Means with superscripts within the same row significantly differ at p < 0.05. n = 8*.

*Con, basal diet; CB, basal diet + 0.05% CB; XOS, basal diet + 0.02% XOS; CB + XOS, basal diet + 0.05% CB + 0.02% XOS; ADFI, average daily feed intake; ADG, average daily gain; BW, body weight; F:G, feed intake/body weight gain*.

### Carcass Trait

As shown in Table 3, at day 56 of the trial, dietary CB or XOS addition decreased (*p* < 0.05) the preslaughter weight; CB and CB + XOS groups were lower (*p* < 0.05) in the carcass weight, carcass yield, and skin thickness compared with the Con group. In addition, pigs fed with CB + XOS had a highest (*p* < 0.05) backfat thickness. Pigs fed with XOS had a higher (*p* < 0.05) carcass yield and backfat thickness at day 84 of the trial compared with fed with those Con diet. The CB, XOS, and CB + XOS groups were higher in the backfat thickness at day 84 of the trial compared with the Con group.

**Table 3:** Effects of dietary addition with CB and XOS on carcass traits of Huanjiang mini-pigs.

**Items**	**Con group**	**CB group**	**XOS group**	**CB + XOS group**	**SEM**	***p* value**
**Day 28 of the trial**						
Pre-slaughter weight, kg	14.97	14.92	15.49	14.80	0.42	0.76
Carcass weight, kg	8.04	7.59	8.13	7.61	0.35	0.57
Carcass yield, %	53.99	53.24	51.20	52.36	0.59	0.26
Total skin, %	24.45	22.32	22.84	23.93	0.49	0.14
Total bone, %	18.59	20.10	18.14	18.81	0.54	0.39
Total skeletal muscle, %	35.98	37.94	37.91	34.40	0.60	0.06
Total fat, %	20.76	18.87	21.11	21.89	0.62	0.27
Loin eye area, cm^2^	5.88	6.86	7.24	7.62	0.48	0.12
Skin thickness, mm	1.79	1.59	2.13	2.06	0.26	0.22
Backfat thickness, mm	12.81	11.69	11.50	10.93	0.48	0.26
**Day 56 of the trial**						
Pre-slaughter weight, kg	28.57^a^	24.94^c^	26.21^bc^	27.39^ab^	0.46	<0.01
Carcass weight, kg	16.67^a^	13.61^b^	15.45^ab^	14.50^b^	0.46	0.01
Carcass yield, %	58.20^a^	54.40^b^	57.19^ab^	54.52^b^	0.62	0.04
Total skin, %	19.77^b^	19.54^b^	21.90^a^	18.60^b^	0.44	<0.01
Total bone, %	14.28^b^	20.39^a^	15.84^b^	19.28^a^	0.62	<0.01
Total skeletal muscle, %	33.62	31.58	31.30	32.13	0.81	0.82
Total fat, %	31.20	27.66	29.57	29.99	0.64	0.22
Loin eye area, cm^2^	6.90	5.97	7.08	6.60	0.44	0.51
Skin thickness, mm	3.22^a^	2.13^b^	2.44^b^	2.03^b^	0.26	<0.01
Backfat thickness, mm	21.88^b^	19.66^b^	21.53^b^	25.60^a^	0.63	<0.01
**Day 84 of the trial**						
Pre-slaughter weight, kg	40.70	39.83	40.78	39.86	0.63	0.89
Carcass weight, kg	24.45	23.72	25.40	24.68	0.49	0.39
Carcass yield, %	59.32^b^	59.54^b^	62.34^a^	58.94^b^	0.56	0.04
Total skin, %	19.09^b^	20.55^b^	19.92^b^	25.24^a^	0.61	<0.01
Total bone, %	14.80	13.23	14.63	13.16	0.50	0.24
Total skeletal muscle, %	29.69	29.75	31.03	29.31	0.66	0.78
Total fat, %	35.06	36.46	36.95	33.50	0.74	0.42
Loin eye area, cm^2^	10.08	10.92	12.25	11.43	0.50	0.20
Skin thickness, mm	4.94^a^	2.45^b^	2.58^b^	2.60^b^	0.26	<0.01
Backfat thickness, mm	27.42^c^	37.53^a^	32.41^b^	34.26^ab^	0.76	<0.01

*Means with superscripts within the same row significantly differ at p < 0.05. n = 8*.

*Con, basal diet; CB, basal diet + 0.05% CB; XOS, basal diet + 0.02% XOS; CB + XOS, basal diet + 0.05% CB + 0.02% XOS*.

### Meat Quality

As shown in Table 4, CB, XOS, and CB + XOS groups were higher (*p* < 0.05) in the pH_45min_ and were lower (*p* < 0.05) in the marbling score at day 28 of the trial compared with the Con group. The CB + XOS group was higher (*p* < 0.05) in the a^*^ value at day 56 of the trial compared with the XOS group. At day 84 of the trial, higher (*p* < 0.05) a^*^ value and marbling score were observed in CB and XOS groups compared with the Con group. Dietary addition with CB and/or XOS decreased (*p* < 0.05) the L^*^ value in muscle.

**Table 4:** Effects of dietary addition with CB and XOS on meat quality of Huanjiang mini-pigs.

**Items**	**Con group**	**CB group**	**XOS group**	**CB + XOS group**	**SEM**	***P* value**
**Day 28 of the trial**						
Color measurements						
L* value	44.19	48.62	44.66	44.08	0.75	0.16
a* value	20.23	21.72	20.73	20.42	0.63	0.78
b* value	6.06	6.45	6.10	6.90	0.36	0.33
pH_45min_	5.71^c^	6.37^a^	6.10^b^	6.06^b^	0.16	<0.01
Marbling score	3.00^a^	1.75^c^	2.00^bc^	2.38^b^	0.27	<0.01
**Day 56 of the trial**						
Color measurements						
L* value	43.74	44.22	43.84	42.03	0.59	0.41
a* value	17.25^ab^	18.36^ab^	16.48^b^	19.18^a^	0.50	0.05
b* value	4.61	4.68	4.39	4.18	0.30	0.49
pH_45min_	6.12	6.26	6.17	6.15	0.17	0.68
Marbling score	1.88	1.63	2.00	1.75	0.22	0.27
**Day 84 of the trial**						
Color measurements						
L* value	43.28^a^	40.23^b^	40.37^b^	40.89^b^	0.42	<0.01
a* value	16.34^b^	18.90^a^	19.48^a^	17.30^b^	0.41	<0.01
b* value	4.71	5.37	5.29	4.91	0.31	0.28
pH_45min_	6.40^a^	6.25^a^	5.25^b^	6.27^a^	0.23	<0.01
Marbling score	3.50^b^	4.25^a^	4.38^a^	4.00^ab^	0.8	0.04

*Means with superscripts within the same row significantly differ at p < 0.05. n = 8*.

*Con, basal diet; CB, basal diet + 0.05% CB; XOS, basal diet + 0.02% XOS; CB + XOS, basal diet + 0.05% CB + 0.02% XOS*.

### Chemical Composition of Muscle

As shown in Table 5, pigs fed with CB or XOS had lower (*p* < 0.05) contents of Glu, Ile, Leu, Met, Tyr, Val, and essential amino acid (EAA) in LD muscle at day 28 of the trial compared with the Con group. At day 56 of the trial, XOS group was higher (*p* < 0.05) in the contents of His, Lys, Met, Phe, and EAA compared with the Con group. At day 84 of the trial, dietary CB addition increased (*p* < 0.05) the contents of Asp, Met, Pro, Ser, Tyr, Val, nonessential amino acid (NEAA), and total amino acid (TAA) and XOS addition increased (*p* < 0.05) the contents of CP, Ala, Arg, Asp, Glu, Ile, Leu, Lys, Met, Pro, Ser, Thr, Tyr, Val, EAA, flavor amino acid (FAA), NEAA, and TAA in LD muscle compared with the Con group.

**Table 5:** Effects of dietary addition with CB and XOS on crude protein (CP) content and amino acid composition of *longissimus dorsi* muscle of Huanjiang mini-pigs (100 g fresh muscle).

**Items**	**Con group**	**CB group**	**XOS group**	**CB + XOS group**	**SEM**	***p* value**
**Day 28 of the trial**						
CP	17.49	17.30	17.29	17.76	0.30	0.53
**EAA**						
His	0.85^a^	0.76^b^	0.81^ab^	0.85^a^	0.08	0.02
Ile	0.99^a^	0.91^b^	0.93^b^	0.95^b^	0.06	<0.01
Leu	1.63^a^	1.51^b^	1.54^b^	1.58^ab^	0.09	<0.01
Lys	1.80	1.70	1.72	1.78	0.10	0.10
Met	0.52^a^	0.46^c^	0.47^bc^	0.51^ab^	0.07	0.01
Phe	0.82	0.77	0.78	0.82	0.08	0.14
Thr	0.90	0.85	0.86	0.89	0.07	0.09
Val	1.05^a^	0.98^b^	0.99^b^	1.02^ab^	0.07	0.02
**NEAA**						
Ala	1.12	1.08	1.07	1.10	0.07	0.19
Arg	1.28	1.24	1.24	1.28	0.08	0.33
Asp	1.83	1.75	1.77	1.82	0.10	0.19
Glu	2.93^a^	2.77^b^	2.79^b^	2.89^ab^	0.12	0.05
Gly	0.87	0.95	0.90	0.92	0.08	0.06
Pro	0.72	0.74	0.72	0.72	0.06	0.56
Ser	0.73	0.70	0.70	0.72	0.06	0.17
Tyr	0.67^a^	0.61^b^	0.63^b^	0.64^ab^	0.06	<0.01
EAA	8.55^a^	7.95^c^	8.11^bc^	8.38^ab^	0.21	0.01
NEAA	10.01	9.65	9.82	10.10	0.25	0.34
FAA	8.03	7.80	7.78	8.00	0.20	0.30
TAA	18.55	17.60	17.94	18.49	0.31	0.08
**Day 56 of the trial**						
CP	19.08^b^	18.85^b^	19.47^b^	20.27^a^	0.27	<0.01
**EAA**						
His	0.88^c^	0.92^c^	0.98^b^	1.04^a^	0.07	<0.01
Ile	1.05^b^	1.06^b^	1.11^ab^	1.14^a^	0.08	<0.01
Leu	1.69^b^	1.72^b^	1.77^ab^	1.83^a^	0.10	0.01
Lys	1.93^c^	1.95^bc^	2.03^ab^	2.11^a^	0.11	<0.01
Met	0.53^b^	0.57^ab^	0.60^a^	0.59^a^	0.070	0.01
Phe	0.89^b^	0.89^b^	0.95^a^	0.99^a^	0.07	<0.01
Thr	0.95^b^	0.98^b^	1.00^ab^	1.04^a^	0.08	0.01
Val	1.10^b^	1.10^b^	1.15^b^	1.20^a^	0.08	<0.01
**NEAA**						
Ala	1.14^b^	1.17^ab^	1.17^ab^	1.22^a^	0.08	0.04
Arg	1.36^b^	1.38^b^	1.42^ab^	1.47^a^	0.09	0.02
Asp	1.94^b^	1.99^b^	2.04^ab^	2.12^a^	0.11	<0.01
Glu	3.07	3.16	3.22	3.31	0.15	0.07
Gly	0.90^c^	0.95^ab^	0.93^bc^	0.99^a^	0.07	<0.01
Pro	0.73	0.75	0.76	0.78	0.07	0.06
Ser	0.75^b^	0.78^ab^	0.78^ab^	0.81^a^	0.07	0.04
Tyr	0.69^b^	0.70^b^	0.73^ab^	0.75^a^	0.070	0.01
EAA	9.11^b^	9.19^b^	9.58^a^	9.94^a^	0.21	<0.01
NEAA	10.85^b^	10.86^b^	11.03^ab^	11.46^a^	0.24	0.04
FAA	8.51^b^	8.66^b^	8.77^ab^	9.12^a^	0.21	0.02
TAA	19.96^b^	20.06^b^	20.61^ab^	21.40^a^	0.31	<0.01
**Day 84 of the trial**						
CP	18.87^b^	19.49^ab^	19.77^a^	18.94^b^	0.27	0.01
**EAA**						
His	0.96	1.00	1.01	0.95	0.08	0.05
Ile	1.05^b^	1.09^ab^	1.13^a^	1.06^b^	0.08	0.03
Leu	1.71^b^	1.78^ab^	1.83^a^	1.72^b^	0.10	0.03
Lys	1.94^b^	2.01^ab^	2.08^a^	1.96^b^	0.11	0.04
Met	0.51^b^	0.59^a^	0.58^a^	0.52^b^	0.06	<0.01
Phe	0.89	0.90	0.93	0.88	0.07	0.08
Thr	0.97^b^	1.00^ab^	1.03^a^	0.96^b^	0.07	<0.01
Val	1.15^b^	1.21^a^	1.23^a^	1.13^b^	0.07	<0.01
**NEAA**						
Ala	1.17^b^	1.21^ab^	1.24^a^	1.19^b^	0.08	0.04
Arg	1.36^b^	1.42^ab^	1.45^a^	1.37^b^	0.09	0.03
Asp	2.00^b^	2.09^a^	2.13^a^	1.97^b^	0.09	<0.01
Glu	3.09^b^	3.21^ab^	3.32^a^	3.12^b^	0.14	0.08
Gly	0.92	0.94	0.96	0.90	0.08	0.14
Pro	0.75^b^	0.79^a^	0.79^a^	0.76^ab^	0.06	0.50
Ser	0.76^b^	0.81^a^	0.82^a^	0.77^b^	0.06	<0.01
Tyr	0.69^b^	0.74^a^	0.75^a^	0.69^b^	0.07	<0.01
EAA	9.17^b^	9.59^ab^	9.82^a^	9.18^b^	0.23	<0.01
NEAA	10.74^b^	11.47^a^	11.46^a^	10.82^b^	0.24	<0.01
FAA	8.54^b^	8.87^ab^	9.11^a^	8.55^b^	0.21	<0.01
TAA	19.91^b^	21.06^a^	21.28^a^	20.00^b^	0.31	<0.01

*Means with superscripts within the same row significantly differ at p < 0.05. n = 8*.

*Con, basal diet; CB, basal diet + 0.05% CB; XOS, basal diet + 0.02% XOS; CB + XOS, basal diet + 0.05% CB + 0.02% XOS*.

*Essential amino acid (EAA) = His + Ile + Leu + Lys + Met + Phe + Thr + Val*.

*Nonessential amino acid (NEAA) = Arg + Asp + Ala + Glu + Gly + Pro + Ser + Tyr*.

*Flavor amino acid (FAA) = Ala + Asp + Arg + Glu + Gly*.

*Total amino acid (TAA) = EAA + NEAA*.

As shown in Table 6, at day 28 of the trial, dietary CB or XOS addition decreased (*p* < 0.05) the IMF content in LD muscle; dietary XOS addition or in combination with CB decreased (*p* < 0.05) the percentage of C14:0 in LD muscle compared with the Con group. In addition, dietary XOS and CB addition increased (*p* < 0.05) the percentages of C20:3n-6, C20:4n-6, C22:6n-3, and polyunsaturated fatty acid (PUFA) in LD muscle, whereas decreased (*p* < 0.05) the percentage of monounsaturated fatty acid (MUFA) compared with the Con group. At day 56 of the trial, the IMF content in LD muscle was lower (*p* < 0.05) and the C18:3n-6 percentage was higher (*p* < 0.05) in the CB and XOS groups compared with the Con group. The CB + XOS group was higher (*p* < 0.05) in the percentage of C18:3n-6 compared with the Con group. At day 84 of the trial, the percentages of C20:1 was lower (*p* < 0.05) in the CB group compared with the Con group.

**Table 6:** Effects of dietary addition with CB and XOS on intramuscular fat (IMF) content and fatty acid composition of *longissimus dorsi* muscle of Huanjiang mini-pigs (% total fatty acids).

**Items**	**Con group**	**CB group**	**XOS group**	**CB + XOS group**	**SEM**	***p* value**
**Day 28 of the trial**						
IMF, g/100 g fresh muscle	6.50^a^	3.83^b^	3.80^b^	6.72^a^	0.44	<0.01
C14:0	1.17^a^	1.08^ab^	1.00^b^	0.84^c^	0.13	<0.01
C16:0	25.08	24.95	24.16	23.42	0.41	0.07
C16:1	3.66	3.40	3.44	2.65	0.32	0.10
C17:0	1.16	0.96	0.88	0.94	0.20	0.35
C18:0	13.69	13.57	13.52	14.36	0.28	0.06
C18:1n-9c	29.84	30.65	29.11	25.58	0.72	0.09
C18:2n-6c	17.77	17.05	18.54	19.83	0.59	0.25
C18:3n-6	0.11	0.10	0.11	0.12	0.06	0.59
C20:0	0.21	0.26	0.22	0.22	0.07	0.19
C20:1	0.65	0.64	0.60	0.55	0.14	0.58
C20:3n-6	0.49^b^	0.41^b^	0.43^b^	0.78^a^	0.12	<0.01
C20:4n-6	5.53^b^	4.52^b^	5.75^b^	9.45^a^	0.49	<0.01
C22:6n-3	0.28^b^	0.23^b^	0.29^b^	0.51^a^	0.13	<0.01
SFA^1^	41.32	40.83	39.80	39.77	0.42	0.09
MUFA^2^	34.15^a^	34.69^a^	33.14^a^	27.28^b^	0.72	<0.01
PUFA^3^	24.18^b^	22.31^b^	25.11^b^	30.69^a^	0.76	<0.01
**Day 56 of the trial**						
IMF, g/100 g fresh muscle	4.92^a^	3.36^b^	3.27^b^	4.23^ab^	0.35	0.01
C14:0	1.14	1.02	1.05	1.07	0.16	0.70
C16:0	27.60	26.90	26.56	26.31	0.36	0.11
C16:1	2.43	2.93	2.63	2.67	0.24	0.22
C17:0	0.80^b^	1.06^a^	0.74^b^	0.73^b^	0.12	<0.01
C18:0	15.39	14.65	14.41	14.74	0.39	0.46
C18:1n-9c	31.52	29.28	30.92	30.42	0.69	0.69
C18:2n-6c	14.37	15.56	14.71	16.01	0.64	0.75
C18:3n-6	0.10^b^	0.17^a^	0.14^a^	0.15^a^	0.06	<0.01
C20:0	0.20^b^	0.24^a^	0.20^b^	0.22^ab^	0.06	0.04
C20:1	0.69	0.65	0.66	0.63	0.14	0.92
C20:3n-6	0.48	0.61	0.52	0.57	0.143	0.40
C20:4n-6	4.46	6.06	5.27	5.15	0.45	0.31
C22:6n-3	0.20^ab^	0.22^ab^	0.18^b^	0.24^a^	0.07	0.04
SFA	45.12	43.86	42.96	43.07	0.46	0.06
MUFA	34.63	32.86	34.21	33.72	0.73	0.86
PUFA	19.61	22.61	20.83	22.12	0.78	0.62
**Day 84 of the trial**						
IMF, g/100 g fresh muscle	5.60	4.17	4.83	5.06	0.45	0.40
C14:0	1.25	1.22	1.23	1.31	0.15	0.82
C16:0	26.49	26.65	26.23	26.92	0.38	0.70
C16:1	3.24	3.05	3.21	2.85	0.27	0.54
C17:0	0.60	0.57	0.50	0.60	0.12	0.38
C18:0	13.47	13.92	13.68	14.56	0.33	0.11
C18:1n-9c	36.59	33.72	35.65	33.13	0.61	0.09
C18:2n-6c	11.54	13.54	13.85	12.99	0.57	0.31
C18:3n-6	0.12	0.11	0.12	0.11	0.06	0.91
C20:0	0.23	0.23	0.21	0.26	0.07	0.06
C20:1	0.81^a^	0.66^b^	0.75^a^	0.74^ab^	0.10	<0.01
C20:3n-6	0.12	0.11	0.12	0.11	0.06	0.90
C20:4n-6	0.81	0.65	0.75	0.81	0.13	0.06
C22:6n-3	0.42	0.52	0.56	0.52	0.13	0.18
SFA	3.76	4.68	4.50	4.76	0.43	0.55
MUFA	0.10^b^	0.17^a^	0.18^a^	0.13^b^	0.06	<0.01
PUFA	42.05	42.60	41.85	43.65	0.46	0.15

*Means with superscripts within the same row significantly differ at p < 0.05. n = 8*.

*Con, basal diet; CB, basal diet + 0.05% CB; XOS, basal diet + 0.02% XOS; CB + XOS, basal diet + 0.05% CB + 0.02% XOS*.

*Saturated fatty acids (SFAs) = C14:0 + C16:0 + C17:0 + C18:0 + C20:0*.

*Monounsaturated fatty acids (MUFAs) = C16:1 + C18:1c-9 + C20:1*.

*Polyunsaturated fatty acids (PUFAs) = C18:2n-6c + C18:3n-6 + C20:3n-6 + C20:4n-6*.

## Discussion

The growth performance and meat quality of pigs are influenced by the genotype and rearing conditions (17, 18). This study investigated the effects of dietary CB and XOS addition on growth performance, carcass trait, and meat quality of Huanjiang mini-pigs with different body weight (ages). Our results showed that dietary CB and XOS addition did not alter the final body weight of pigs, which was in line with previous studies, where they reported that dietary CB or XOS addition had no effects on final body weight in the broilers or pigs (19). The XOS cannot be degraded by the digestive enzymes and are metabolized by microbiota in the colon (20). In addition, XOS are competitive inhibitors of cellobiohydrolase I (21), which indicates that dietary addition with XOS may exert a negative effect on growth performance. Here, we found that XOS group showed the highest ADG and lowest F:G ratio at day 28 of the trial. This may be related to the fact that the XOS is sweet and more lure. Previous study also showed that the ADG of pigs fed the XOS diet was higher than pigs fed the Con diet (22). However, we found that dietary CB addition has a negative effect on the F:G during the entire trial period. Chen et al. (23) showed that dietary CB addition had no effect on F:G of piglets; by contrast, Song et al. (24) reported that dietary CB addition could decrease the F:G. These findings suggest that the effect of dietary CB addition on F:G in animals remains highly controversial and further investigations are needed to elucidate this effect. In addition, dietary XOS could ameliorate the negative effects of CB on F:G, which might explain that the XOS are nondigestible oligosaccharides, most of the CB can utilize XOS to produce bioactive metabolites (including SCFA and bioamines), and improve the consumption of intestinal nutrients.

Meat quality is one of the most important economic traits of pigs and can be reflected by the color and pH of the muscles (25). In this study, dietary XOS addition increased carcass yield at day 84 of the trial, but not days 28 and 56 of the trial, indicating that the XOS increased carcass yield in a time-dependent manner. In addition, dietary addition with CB and XOS increased the pH_45min_ value and decreased the L^*^ value at days 56 and 84 of the trial, respectively, suggesting that the meat pH and color were improved by CB and XOS addition. Scheffler et al. (26) reported that inhibition of mitochondrial enzyme activity contributes to accelerated pH decline of meat. The increased pH_45min_ value induced by CB and XOS addition might be due to the improvement of mitochondrial function (27, 28).

It is well documented that IMF or marbling is largely involved in quality and acceptability of meat, particularly meat nutrition, tenderness, juiciness, taste, and conservation ability (29). In this study, no difference was observed in the IMF content between the Con group and the XOS + CB group, suggesting that dietary XOS and CB addition is not a feasible approach to improve meat quality by increasing the IMF content. Meat has a great potential for delivering important nutrients such as proteins, AA, FA, and vitamins (30). The AA content in muscle represents the protein quality of meat. Furthermore, some AA plays key roles in the aroma and flavor profiles of meat. For example, Glu and Asp show a pleasantly fresh taste; Gly, Ala, and Ser present a sweet taste; Arg, Leu, Iso, Val, Phe, Met, and His present a bitter taste; Lys and Pro contribute sweet and bitter tastes; and others show sour or salty tastes (31). In this study, dietary CB and XOS addition increased the muscular contents of CP and AAs at day 56 of the trial. In support of our findings, Liu et al. (32) showed that CB addition increased the contents of EAAs and flavor AAs in muscle. Several previous studies showed that dietary CB and XOS could improve the intestine development (33, 34). Therefore, we suspected that the increased CP and AAs contents in muscle might be due to the enhancement of intestinal absorption ability. The branched-chain amino acids (BCAAs), including Leu, Ile, and Val, are important components of the body proteins and play important roles in regulating cell growth and metabolism. In addition, the BCAA could promote gluconeogenesis and provide energy for body (35). In this study, the BCAAs contents in muscle were increased by dietary XOS addition at day 84 of the trial, suggesting that the nutritional value and flavor of pork are improved by dietary CB and XOS addition.

The FAs also play important roles in flavor generation. However, excessive intake of saturated fatty acid (SFA) would increase the prevalence of cardiovascular diseases. Therefore, reduced SFA percentage and increased unsaturated FA percentage in meat could improve pork quality (36). In this study, dietary CB and XOS addition decreased the percentage of C14:0 and increased the percentages of C18:3n-6, C20:3n-6, C20:4n-6, C22:6n-3, and PUFA in muscle at day 28 of the trial. These findings were in line with Liu et al. (32), who reported that CB addition increased the concentrations of C20:4n-6, C22:6n-3, and PUFA in breast muscle. Therefore, dietary CB and XOS addition might be a feasible strategy for developing high quality meat with healthier lipid contents. The CB is a butyrate-producing probiotic and dietary XOS addition could increase the butyrate concentration in the cecum (22, 37). Butyrate was shown to modulate lipid metabolism (38). These evidences suggest that the changed FA profile in muscle may be due to the butyrate production in pigs fed with CB and XOS. However, the mechanisms require further study.

In conclusion, dietary CB addition improves the meat color and marbling score and increases the contents of Met, Val, Pro, and Try in muscle at day 84 of the trial; dietary XOS addition decreased the values of L^*^ and pH_45min_ and increased the contents of AAs and percentage of MUFA in LD muscle; dietary CB and XOS addition did not alter the growth performance of pigs, but increased the contents of AAs and percentage of PUFA in LD muscle. These findings suggested that dietary CB and/or XOS addition has important complementary advantages for improving pork quality.

## Data Availability Statement

The raw data supporting the conclusions of this article will be made available by the authors, without undue reservation.

## Ethics Statement

The animal study was reviewed and approved by Animal Care and Use Committee of the Institute of Subtropical Agriculture, Chinese Academy of Sciences.

## Author Contributions

XK and XH designed the study. QC, WT, and HJ carried out the animal experiments. QC and MG conducted the sample analysis. QC and CH performed the data collection and analysis. QC, CH, and XK drafted the manuscript. All authors have read and approved the final manuscript.

## Funding

This study was jointly supported by grants from the Special Funds for Construction of Innovative Provinces in Hunan Province (2019RS3022), the Industry and Research Talent Support Project from Wang Kuancheng of the Chinese Academy of Sciences, and the STS Regional Key Project of the Chinese Academy of Sciences (KFJ-STS-QYZD-052).

## Conflict of Interest

The authors declare that the research was conducted in the absence of any commercial or financial relationships that could be construed as a potential conflict of interest.

## Publisher's Note

All claims expressed in this article are solely those of the authors and do not necessarily represent those of their affiliated organizations, or those of the publisher, the editors and the reviewers. Any product that may be evaluated in this article, or claim that may be made by its manufacturer, is not guaranteed or endorsed by the publisher.
